# Agreement of Bioluminescence Measurements and Visual Assessment in Monitoring Occlusal Surfaces of Permanent Teeth

**DOI:** 10.3390/jcm11020464

**Published:** 2022-01-17

**Authors:** Anahita Jablonski-Momeni, Boris Jablonski, Monika Heinzel-Gutenbrunner, Heike Korbmacher-Steiner

**Affiliations:** 1 Department of Orthodontics, Dental School, Marburg Medical Faculty, Philipps-University Marburg, 35039 Marburg, Germany; korbmacher@med.uni-marburg.de; 2 Dental Practice, 35457 Lollar, Germany; jaboris112@yahoo.de; 3 MH Statistik, Statistical Services, 35041 Marburg, Germany; monika.heinzel@mh-statistik.com

**Keywords:** enamel caries, active lesion, bioluminescence, monitoring, occlusal surface

## Abstract

Background: Caries lesion activity is typically assessed by visual–tactile criteria. Regular monitoring is required to detect the transition of lesions and to ensure that the initial assessment was valid. This clinical study aimed to evaluate the agreement of bioluminescence measurements (Calcivis imaging system, Cis) with visual examination to assess caries lesion activity and to monitor occlusal surfaces. Methods: The occlusal surfaces of ninety-one permanent posterior teeth were assessed for the presence or absence of active caries lesions with ICCMS criteria and Cis measurements at three visit times: baseline (t1) and six months (t2) and 12 months (t3) after baseline. Results: At the baseline visit, 70% of the included occlusal sites were assessed visually as active lesions (ICCMS codes 1 and 2). At t3, 64.8% of the sites showed signs of an active lesion. The percentage agreements between the visual and Cis methods were 87.8% (t1), 89.9% (t2) and 88.6% (t3). The corresponding κ-values were 0.71 (95% CI 0.52;0.87), 0.75 (95% CI 0.59;0.89) and 0.77 (95% CI 0.61;0.90), respectively. No significant difference between the visual and bioluminescence systems was found at any visit (*p* > 0.05). The results based on cluster randomization (generalized estimation equations) showed no significant differences between the visual and Cis findings for all visits (*p* = 0.108, Wald Χ^2^ with 1 df = 2.587). Conclusion: The bioluminescence system demonstrated substantial agreement for the activity assessment of occlusal lesions compared to the findings obtained by visual assessment over twelve months.

## 1. Introduction

Caries lesions can be categorized according to their anatomical location on the tooth; their severity; their depth of penetration into the tissue; and their activity status, such as being active or inactive [1]. The activity status of a caries lesion is defined by surface characteristics, such as change in texture, translucency and color, and other factors, such as the presence of thick plaque and a plaque stagnation area, as well as gingivitis discriminating the likelihood of a lesion progressing or non-progressing/arrested [1]. Methods aiming at the determination of lesion activity are normally based on visual or visual–tactile criteria [2].

There are reports of using optical devices for the detection and quantification of noncavitated early enamel lesions [3] as a tool for the documentation and monitoring of lesions. Such methods can support the diagnostic process in quantifying and/or visualizing caries. A new approach aims to distinguish between active and inactive lesions based on bioluminescence measurements. The Calcivis^®^ Imaging System (Calcivis Ltd., Edinburgh, UK) is a novel method based on the bioluminescence technique for detecting demineralization in enamel. The bioluminescence technique consists of applying a specific calcium-sensitive photoprotein to the tooth surface. The photosensitive protein binds to free calcium ions, which are released during the demineralization of enamel [4,5], and a light signal is emitted upon binding to solvated calcium ions. If the tooth is undergoing net demineralization, a light signal can be observed [6]. Such luminescence areas are typically present as blue spots, demonstrating the presence of demineralized surfaces. In a preliminary study on occlusal caries lesions, the bioluminescence system demonstrated high reproducibility and good diagnostic accuracy values for the assessment of active caries lesions in vitro based on histological findings [7]. Moreover, the ability of the bioluminescence technology to identify demineralization on smooth surfaces adjacent to orthodontic brackets was shown [8]. In a recently published paper, the bioluminescence system could differentiate tooth surfaces clinically identified as involving active enamel lesions from sound sites [9].

Part of the modern caries diagnosis concepts based on tooth preservation and patient-level prevention is the monitoring and reassessment of clinically sound tooth surfaces or those with initial lesions that do not require invasive treatment [10,11]. A patient’s risk level is derived from social, medical, behavioral (oral hygiene, diet etc.) and past dental histories, together with an oral examination [11]. This includes the identification of active lesions and enables the development of a personalized care plan leading to different management options and the determination of recall intervals for active and inactive lesions.

The assessment of a lesion’s true activity status in a single visit remains challenging, and the determination of which initial lesions will progress can normally be validated prospectively by serial monitoring over time [9,12]. It was shown that the bioluminescence method can be used to visualize active demineralization sites in clinically identified enamel lesions [9]. Since the ability of the device for monitoring purposes has not yet been under study, the present study aimed to evaluate the agreement of the bioluminescence method to monitor caries lesions compared with visual findings.

## 2. Materials and Methods

This prospective, non-interventional study was conducted ethically in accordance with the Word Medical Association Declaration of Helsinki. The study protocol was reviewed and approved by the Ethics Committee of the Medical Faculty of the Philipps-University of Marburg, Germany (approval number 156/19, date of approval: 11 November 2019). The study was registered in the German Clinical Trials Register (DRKS00020123, date of registry: 3 December 2019).

**Figure 1:**
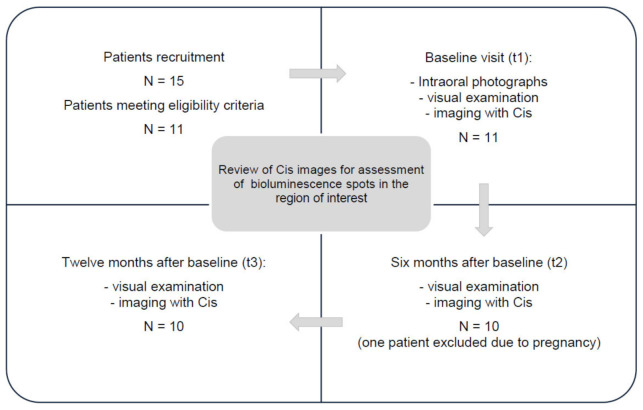
Outline of the study design.

The outline of the study procedure is displayed in Figure 1.

### 2.1. Sample Size Calculation

A sample size calculation was performed using the software PASS 11, NCSS, version 11.0.7 (Kaysville, UT, USA), based on data of a preliminary in vitro study assessing the agreement between visual and bioluminescence measurements and diagnostic accuracy of activity assessment [7]. A total of 48 occlusal surfaces were calculated for a power of 0.95 and α = 0.05. To compensate for the cluster effect that would result from the fact that teeth from the same patient are considered as dependent, the calculated number of cases was increased by 20%. Adding a dropout rate of 10%, 64 occlusal surfaces were calculated for inclusion in the examination.

### 2.2. Examiner Background and Training

Two examiners were involved in the clinical examinations. One examiner (A.J.M.) is a dentist with a background in cariology and is experienced in the use of ICCMS (International Caries Classification and Management System) and several other detection systems. The other examiner (B.J.) is a dentist with experience in the use of ICCMS based on his dental educational background [13] and is experienced as an examiner in other caries detection studies [14].

Prior to the study, the examiners were trained in the use of the bioluminescence technology by a representative of the manufacturer.

To ensure quality of data assessment, intra- and inter-examiner agreements (κ-values) were evaluated prior to the clinical examinations. Occlusal surfaces of 50 extracted human posterior teeth were independently examined twice within one week using ICCMS criteria for lesion extent and activity assessment. Since the extracted teeth were cleaned prior to the examinations, the presence of plaque was not included in the criteria for activity assessment. Bioluminescence measurements of the teeth were produced with an in vitro device, and the images were evaluated for presence or absence of bioluminescence spots. The κ-values are summarized in Table 1, all corresponding to an almost perfect agreement [15].

### 2.3. Participants

The participants were recruited between November 2019 and January 2020 at a dental practice where the examinations took place. Eleven patients were included for participation in the study, and written informed consent was obtained. One participant was excluded during the study due to pregnancy. In total, 5 female and 5 male patients with a mean age of 34 years (23–48 years) took part in the study until the final examinations. Examinations took place between January 2020 and February 2021. All patients lived in an area with up to 0.25 mg F^−^/L in the tap water, which has been constant for many years. According to the data of a representative national survey, the mean number of teeth with active initial lesions was reported as 0.7 and for inactive lesions as 0.8 for the whole country [16].

The inclusion criteria were the following: age ≥18 years; signed, written consent before participation in the study; patients who were willing to attend all study-related appointments; patients with at least 6 posterior teeth without extended restorations; and patients with at least one initial caries lesion on the occlusal surface of posterior teeth.

Exclusion criteria were the following: patients with hypersensitivity or allergy to proteins (no separate allergy testing was planned for the study); pregnant women (no separate pregnancy test was planned for the study); patients with teeth after bleaching (<2 weeks before measurements); patients with acute toothache; occlusal surfaces with extensive carious lesions (visible dentin caries); teeth with developmental defects, fluorosis and other opacities or erosive wear.

### 2.4. Clinical Examinations

First, a visual assessment was carried out by one examiner in order to check for thick plaque areas. Then, the teeth were cleaned using a rotating brush with a prophylactic paste (Cleanic Prophy-Paste with RDA 27, Kerr GmbH, Biberach, Germany) and dental floss. Teeth were rinsed and dried using a 3-in-1 syringe and isolated with cotton rolls. A mirror and a ball-ended probe were used for visual examination under optimal light conditions in the dental chair. No magnification glasses were used.

The findings (lesion staging and activity assessment) were documented based on the ICCMS criteria for occlusal surfaces [17]. The staging was performed according to the following criteria:

Sound tooth surfaces (code 0): no evidence of visible caries when viewed clean and after 5 s of air drying;

Initial stage caries (codes 1 and 2): first or distinct visual changes in enamel seen as a carious opacity or visible discoloration (white spot lesion and/or brown carious discoloration) not consistent with clinical appearance of sound enamel, with no evidence of surface breakdown or underlying dentine shadowing.

For lesion activity assessment, the following characteristics were considered:

Signs of active lesions: surface of enamel is whitish/yellowish, opaque with loss of luster and feels rough when the tip of the ball-ended probe is moved gently across the surface. Lesion is in a plaque stagnation area. The lesion may be covered by thick plaque prior to cleaning.

Signs of inactive lesions: surface of enamel is whitish, brownish or black. Enamel may be shiny and feels hard and smooth when the tip of the ball-ended probe is moved gently across the surface. Lesion may not be covered by thick plaque prior to cleaning.

Dental images were taken from the teeth to relocate the regions of interest at a later time (dentaleyepad, doctorseyes GmbH, Ochsenhausen, Germany). All examinations were performed at three times: baseline (t1) and six months (t2) and twelve months (t3) after baseline.

### 2.5. Examination with the Bioluminescence Device Calcivis Imaging System (Cis)

After visual examination, Cis measurements were performed by another examiner independently. The Cis consisted of an intraoral device connected to a laptop with specific software (CALCIVIS application software); single-use sterile device syringes; vial adaptors; and single-use applicators. All steps were performed according to the manufacturer’s instructions. Prior to the image taking, the photoprotein was prepared and was filled in a syringe. The syringe was then inserted into the device. With each syringe filling, 5 images could be produced. At the beginning of each examination day, a function test was run using a special kit supplied with the system. An image was produced from a deposit of calcium chloride available in the kit. Once a bioluminescence image was displayed on the screen, the system was confirmed to function as intended.

**Figure 2:**
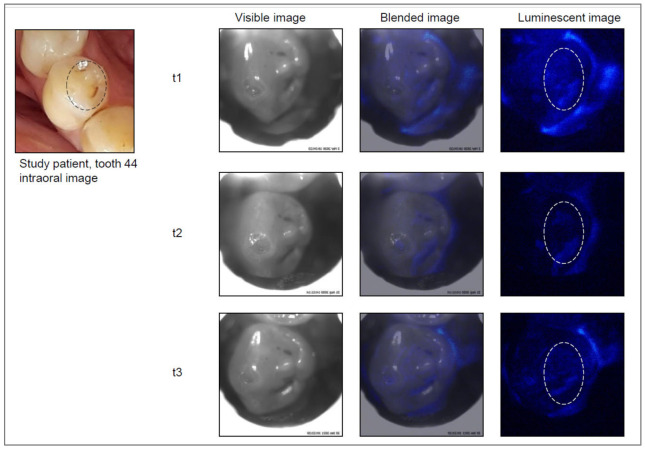
Example of a study tooth (lower right premolar) with an active lesion at all visits: t1 = baseline, t2 = six months after baseline, t3 = twelve months after baseline.

**Figure 3:**
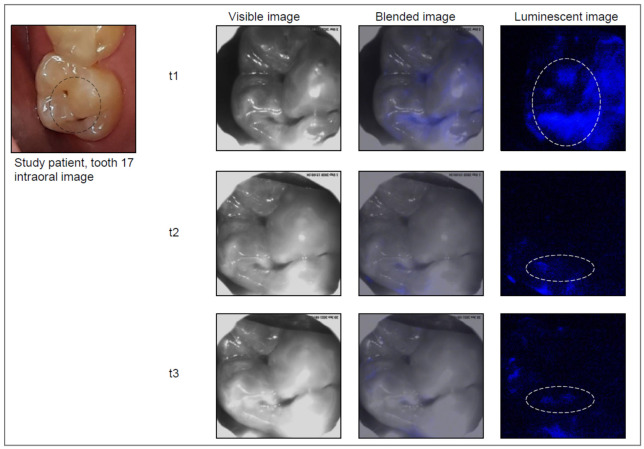
Example of a study tooth (upper right molar) with an active lesion at all visits. The bioluminescence signal was lower at t2 and t3 but still visible.

**Figure 4:**
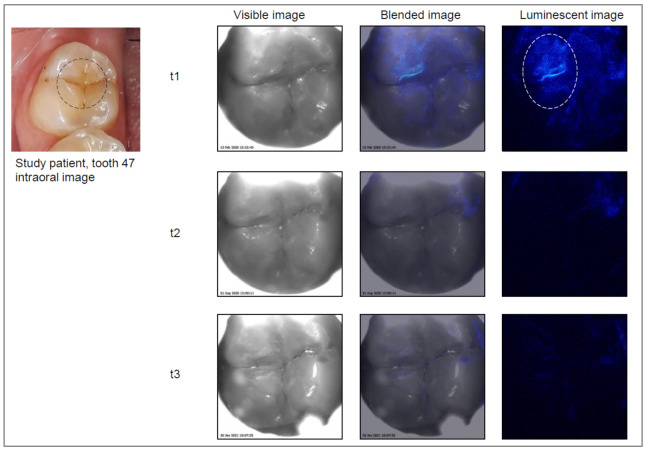
Example of a study tooth (lower right molar) with an active lesion at t1. At t2 and t3, the tooth was assessed visually as inactive. No bioluminescence signal is visible on the images at t2 and t3.

**Figure 5:**
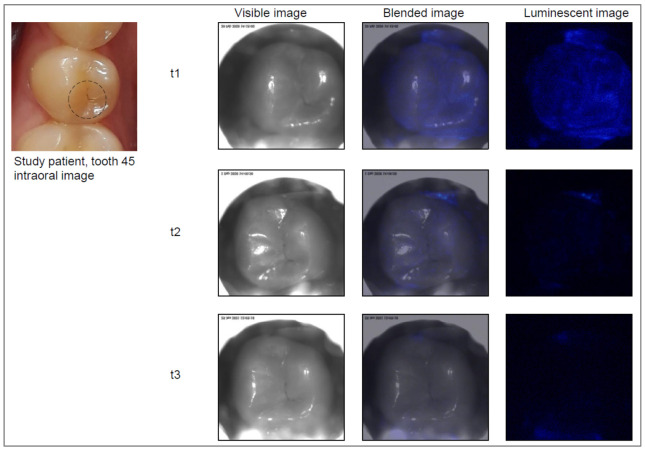
Example of a study tooth (lower right premolar) with an inactive lesion. At t1, a bioluminescence signal was visible all over the occlusal surface, whereas at t2 and t3, no signal was detectable in the fissure.

Care was given to ensure that the tooth surface and surrounding area were free from saliva using cotton rolls and saliva ejectors. Each tooth was then air dried for 5–10 s. The head of the device was placed on the occlusal surface, and the image capture procedure was started. An image of the chosen tooth was first captured as a visible, black and white, light image, and then the photoprotein was applied, followed by capturing the luminescence signal. This automated process was performed within less than 0.5 s, including automated photoprotein application. Images were stored digitally on the laptop. The software overlaid the two sets of images (black and white and luminescence), resulting in an “active demineralization map” of each imaged tooth, highlighting locations where elevated levels of calcium ions were present (Figure 2, Figure 3, Figure 4 and Figure 5).

The participant rinsed out their mouth with non-fluoridated tap water after imaging was completed. Any adverse events were recorded at the visits.

All Cis measurements were performed in the same appointment right after the visual examination.

After the examinations, each patient received oral care instructions from a dental hygienist as part of the practice-based prevention program, and fluoride gel (Lunos^®^ Fluoridgel, Dürr Dental SE, Bietigheim-Bissingen, Germany) containing 12,300 ppm sodium fluoride was applied to the teeth. This gel is commonly used for topical fluoridation in the dental office where the study was performed, and it is regularly used after professional tooth cleaning and for patients with moderate and high caries risk.

### 2.6. Evaluation of Cis Images

The Cis images were classified independently after the examinations. For this purpose, a region of interest (ROI) was drawn on the visible image based on the clinical examination (Figure 2, Figure 3, Figure 4 and Figure 5). Then, the corresponding Cis image was displayed on the screen, and the ROI was investigated for presence or absence of luminescent spots as a yes/no decision.

### 2.7. Statistical Analysis

The statistical evaluation was performed using IBM^®^ SPSS ^®^ Statistics Software Version 27 (IBM Deutschland GmbH, Ehningen, Germany).

The distribution of the findings (visual, Cis) was represented using cross-tabulation. Kappa statistic (unweighted kappa) was calculated to compare the findings (based on yes/no decision for activity assessment), and the McNemar test was used to evaluate the differences. Additionally, generalized estimation equations (GEEs) were modeled in order to evaluate the difference between the visual and bioluminescence findings in a multilevel model, which considered that the data were obtained by a cluster randomization. In the multilevel model, the patient was determined as level-1 unit, while the teeth were defined as level-2 unit and the visit time as level-3 unit. Additionally, both visual and bioluminescence methods were considered to be used for the same tooth.

Areas under the ROC curve, sensitivity and specificity values of Cis were calculated using the visual assessment as the clinical reference.

The level of significance was set at α = 0.05.

## 3. Results

The occlusal surfaces of 91 permanent posterior teeth of 10 patients were included in the examination. Of these, 36 (39.6%) were premolars and 55 (60.4%) were molars. The distribution of all findings at each visit is displayed in Table 2. The percentages of agreement between visual and bioluminescence methods were 87.8% at visit 1, 89.9% at visit 2 and 88.6% at visit 3. The corresponding κ-values for all teeth were between 0.71 and 0.77, corresponding to a substantial agreement.

No significant differences were found between the findings at each visit (*p*-values > 0.05, McNemar test) (Table 2).

At the first visit, 70.0% of the occlusal surfaces were visually assessed as active lesions. At the last visit after one year, 64.8% of the teeth were assessed with signs of active lesions (Table 2). These changes were evaluated by visual and Cis findings without any significant difference (*p* = 0.069, McNemar test).

The results of the GEE, taking into account the clustered nature of the data, showed no significant differences between the visual and bioluminescence findings for all visits (*p* = 0.108, Wald Χ^2^ with 1 df = 2.587).

Areas under the ROC curve, sensitivity and specificity values of the Cis based on visual activity assessment are presented in Table 3.

At the end of the study visits, none of the initial lesions progressed toward a moderate or severe lesion. The number of sites with transition toward active or inactive lesions between the first and last study visit is summarized in Table 4. Based on the findings at the last study visit, none of the lesions were planned to receive restorative treatment. The patients were scheduled for further recall appointments based on the decision of their dentist.

## 4. Discussion

In the present in vivo study, a bioluminescence device (Calcivis imaging System, Cis) was used to monitor the occlusal surfaces of permanent teeth, and the findings were compared with visual assessments based on ICCMS criteria for lesion activity assessment [17]. The Cis measurements were not significantly different from the values obtained in the visual assessment, confirming the results of previous in vitro studies [7].

Caries lesion activity assessment seeks to differentiate lesions deemed caries active from lesions deemed caries inactive in order to provide optimal care planning where there is a focus on arresting active lesions [1]. In a policy statement on caries lesions and first restorative treatment by The World Dental Federation (FDI), it is recommended that any tissue removal decision must consider both lesion stage and activity issue, the patient’s condition and caries risk and aesthetic demands [18]. An active lesion is considered to have a greater likelihood of transition (progress, arrest or regress) than an inactive lesion. However, such changes can normally be observed longitudinally to be sure that the initial assessment was valid [9], such as how it was performed in the present study.

Different methods have been developed for the activity assessment of coronal carious lesions in clinical settings [2] to take into account the dynamic nature of dental caries. The most widespread methods are systems based on combinations of visual (appearance and plaque stagnation) and tactile criteria. However, devices based on fluorescence or bioluminescence were also reported to be used for caries lesion activity assessment, with a deficiency of existing validation data compared to visual–tactile systems [2]. As an example, a laser fluorescence method could not differentiate between active and inactive initial caries lesions on occlusal surfaces [19]. However, it was shown that by measuring specular reflection and roughness, it was possible to distinguish sound and demineralized/active stages of caries lesions in vitro [20]. Measuring red fluorescence was also suggested as a useful way of objectively evaluating lesion activity of smooth-surface lesions [21].

The recently introduced digital imaging system Calcivis was developed as a bioluminescent marker to illuminate the assessment of dental caries and dental erosion through its binding of calcium ions, known to be released during the process of demineralization. In vitro and clinical studies have already been performed on occlusal and smooth surfaces [6,7,8,9], showing the ability of the bioluminescence method to visualize active demineralization and enamel erosion. However, Drancourt et al. [22] concluded in an in vitro study that it was difficult to confirm the ability of a bioluminescence-based camera and a fluorescence-based system for the activity assessment of coronal carious lesions. The authors reported lower sensitivity and specificity values for the bioluminescence system compared to the fluorescence device. The findings were based on a visual assessment of the teeth as a reference, so the results cannot be compared to the diagnostic validity data published in another in vitro study [7], where high diagnostic accuracy (area under the curve 0.91) was reported for the bioluminescence device.

One challenge of using calcium ions on the tooth surface as a marker for current mineral loss could be the circumstance that inactive lesions may also lose calcium temporarily, e.g., after the consumption of an erosive drink. An accurate distinction between erosive tooth wear and an active lesion is necessary to avoid false positive measurements.

A major methodological challenge when comparing the visual and bioluminescence system is that this new technology operates at a molecular/ionic level, independent of the macro-morphology of the enamel, whereas the reference standard (i.e., visual assessment) operates at a macromorphological level [9]. Such methodological problems basically apply to studies that compare different systems with one another, and for which there is no 100% true gold standard available. Further research in this direction is therefore necessary in order to develop a suitable gold standard. However, in a previous in vitro study, the high agreement between both methods was evaluated, demonstrating that a comparison of the bioluminescence findings to those of the visual assessment was possible to some extent [7].

In the present in vivo study, we calculated the sensitivity and specificity to compare the results with other studies. Typically, a gold standard, such as histological findings, is needed. Due to the clinical nature of our study, and because no teeth were scheduled for extraction, we could only calculate sensitivity and specificity based on the visual findings. In a previous in vitro study with histology as the gold standard, the bioluminescence system showed a good result in the diagnostic accuracy, comparable to the visual assessment of active vs. inactive lesions [7]. The findings of laboratory-based studies were used as a basis for the preparation of the present monitoring study. Here, the findings were not only based on each single visit. Moreover, the clustered nature of the data was considered by a statistical model. Even when using such a multilevel model, no difference between the visual and Cis findings was observed.

Despite the good results, some shortcomings occur when using the technology. One shortcoming is the lack of any scale to quantify the luminescence areas. The decision about the activity of a lesion is given as a yes/no answer, which is comparable to the visual assessment about lesion activity. In order to follow up active lesions and have an idea as to whether the free calcium ions have reduced at different recall intervals, a categorical scale would be helpful as in the case of some fluorescence-based systems. In a recently published study by Jablonski-Momeni et al. [8], an attempt was made to quantify the luminescence spots by measuring the pixels in the areas of interest. However, the use of software was necessary, and it would be easier if such quantification could be integrated in the already existing Cis software for clinical use.

In the clinical situation, we experienced that the placing of the camera head was sometimes challenging, especially on the occlusal surfaces of premolar teeth. However, the results showed comparable values for agreement between the visual and Cis findings for both premolars and molars (Table 2), indicating no significant issues in the process of image taking for either type of tooth. Generally, the process of digital image taking takes more time than visual examination only. In the present study, the Cis measurements took about 15–20 min per patient, including the preparation of the device and the protein. This disadvantage is compensated by the fact that the Cis measurements are objective in terms of activity assessment. The use of digital methods for caries activity assessment and the storage of the data allow for a long-term follow-up. The digital images can be stored and called up at any time for the patient. This forms a good basis for patient motivation for oral care and treatment recommendations.

It should also be noted that saliva control plays a very important role while using the Cis. In the case of moisture on the surface, the produced images cannot be evaluated sufficiently. Pitts et al. [9] reported that potential confounders of the Cis include two obvious sources of calcium ions, namely, saliva and the dental biofilm, and these factors can be effectively controlled by isolation, air drying and cleaning, which form part of the Cis pre-imaging protocol. However, such confounders also apply to other caries detection techniques, such as laser fluorescence devices and fluorescence-based cameras.

The assessment of caries lesions at a single appointment is usually not a valid method to recognize the activity status. The likelihood of progression can be identified, but the “real” situation is only seen at the follow-up examination. Caries lesion monitoring is an episodic assessment of the effect of an intervention or the natural behavior on the status of a caries lesion [1], and it was performed in the present study 6 and 12 months after baseline measurements. The agreement between visual and bioluminescence findings at each visit was substantial (Table 2). Moreover, when a multilevel model was calculated, the assessment with both methods showed comparable results over the period of one year. These results would support the ability of the Cis to distinguish active and inactive lesions as valid as visual assessments do over time. The recall intervals were in line with the usual dental check-ups in Germany, which are paid for every six months by health insurances. It is recommended that patients with higher caries risk should have a shorter recall period than patients at lower risk, for monitoring, re-evaluation and the provision of preventive interventions [11]. At the last study visit, 64.8% of the sites still showed signs of lesion activity and would have needed shorter recall intervals. It should be mentioned that after starting the baseline examinations, local restrictions occurred due to the COVID-19 pandemic situation, and it was recommended to restrict dental visits to necessary appointments, such as emergencies. Nevertheless, the main focus of the present study was activity assessment using different methods, and it was not about evaluating the prevention concept or planning restorative treatment. Each patient received professional tooth cleaning and topical and oral care advice at each visit, and, therefore, the six month intervals could be described as adequate, and participation in the study over a somewhat longer period of time was better ensured.

The use of digital technologies in caries activity assessment can overcome the problem of subjective judgment when only visual methods are applied. Moreover, dental professionals will be able to integrate the obtained digital images with clinical findings. This leads to optimized caries management strategies specified for active or inactive lesions. While inactive, noncavitated and cavitated lesions do not require any treatment (except for reasons of form, function or aesthetics), active, noncavitated carious lesions should be managed non- or micro-invasively [23].

## 5. Conclusions

This study was the clinical implantation of in vitro investigations performed on the same systems, i.e., visual and bioluminescence measurements [7]. Although the visual and Cis assessments do not work in the same way (i.e., visual assessment vs. determination of the presence of elevated calcium ion concentrations), comparable results were obtained, not only at one time point but also after monitoring the occlusal sites at three visit times. The use of digital methods for caries activity assessment and the storage of the data allow for a long-term follow-up. Further studies are suggested in order to demonstrate the ability of the Cis to monitor other tooth surfaces at high caries risk, such as on smooth surfaces adjacent to orthodontic brackets.

## Figures and Tables

**Table 1:** Kappa values for examiner agreement prior to the clinical study (95% confidence interval in parenthesis).

	Examiner 1 Intra-Examiner Agreement	Examiner 2 Intra-Examiner Agreement	Inter-Examiner Agreement
ICDAS code	0.88 (0.76;0.99)	0.90 (0.81;1.00)	0.91 (0.81;1.00)
Visual activity assessment	0.84 (0.69;0.98)	0.88 (0.74;1.00)	0.88 (0.75;0.99)
Bioluminescence measurements	0.83 (0.67;0.99)	0.83 (0.67;0.98)	0.96 (0.88;1.00)

**Table 2:** Distribution of visual caries codes cross-tabulated with the results of the bioluminescence measurements. The prefix + or - indicates an active or inactive lesions according to the visual criteria. Fields with agreement between the findings are highlighted in gray.

t1: Baseline Visit	Bioluminescence Measurement			κ-Value for Method Agreement (95% Confidence Interval)	*p*-Value (Difference between Methods)
ICDAS/ICCMS Code	Inactive	Active	N Total		
0	9	1	10 (11.1%)	All teeth: 0.71 (0.52;0.87) Molars: 0.81 (0.56;1.00) Premolar: 0.64 (0.41;0.84)	0.109
1−	8	8	16 (17.8%)		
2−	1	0	1 (1.1%)		
1+	2	37	39 (43.3%)		
2+	0	24	24 (26.7%)		
N total	20 (22.2%)	70 (77.8%)	90 (100%) *		
* At t1, one bioluminescence image was not suitable for evaluation					
t2: 6 months after baseline	Bioluminescence measurement			κ-value for method agreement (95% confidence interval)	*p*-value (difference between methods)
ICDAS/ICCMS code	Inactive	Active	N total		
0	10	0	10 (11.2%)	All teeth: 0.75 (0.59;0.89) Molar: 0.63 (0.34;0.87) Premolar: 0.77 (0.54;0.94)	0.344
1−	16	3	19 (21.3%)		
2−	0	0	0 (0.0%)		
1+	5	30	35 (39.3%)		
2+	1	24	25 (28.2%)		
N total	32 (36.0%)	57 (64.0%)	89 (100%) **		
** At t2, two bioluminescence images were not suitable for evaluation					
T3: twelve months after baseline	Bioluminescence measurement			κ-value for method agreement (95% confidence interval)	*p*-value (difference between methods)
ICDAS/ICCMS code	Inactive	Active	N total		
0	8	2	10 (11.4%)	All teeth: 0.77 (0.61;0.90) Molar: 0.77 (0.51;0.95) Premolar: (0.72 (0.44;0.94)	1.00
1−	17	4	21 (23.8%)		
2−	0	0	0 (0.0%)		
1+	4	28	32 (36.4%)		
2+	0	25	25 (28.4%)		
N total	29 (36.00%)	59 (64.00%)	88 (100%) ***		
*** At t3, three bioluminescence images were not suitable for evaluation					

**Table 3:** Area under the ROC curve (AUC), sensitivity, specificity and likelihood ratios for Cis findings based on visual activity assessment as the reference standard.

Visit	AUC (95% Confidence Interval)	Standard Error	Sensitivity	Specificity	Negative Likelihood Ratio	Positive Likelihood Ratio
1	0.847 (0.745;0.949)	0.05	97.1%	72.4%	0.04	3.52
2	0.889 (0.808;0.970)	0.41	88.5%	89.3%	0.13	8.27
3	0.882 (0.795;0.969)	0.44	93.1%	83.3%	0.08	5.57

**Table 4:** Distribution of visual caries codes cross-tabulated with transition toward active or inactive lesions between baseline visit (t1) and t3.

Transition of the Measurements between t1 and t3	Bioluminescence Measurement			
ICDAS/ICCMS Code	Arrest (from Active to Inactive)	Progression (from Inactive to Active)	No change in Activity Status	N Total
Arrest (from active to inactive)	7	0	0	7 (7.9%)
Progression (from inactive to active)	0	1	1	2 (2.3%)
No change in activity status	5	2	72	79 (89.8%)
N total	12 (13.6%)	3 (3.4%)	73 (83.0%)	88 (100%)

## Data Availability

The data sheet is available from the corresponding author on reasonable request.
